# Strengthening health promotion development with Aboriginal and Torres Strait Islander males in remote Australia: A Northern Territory perspective

**DOI:** 10.1111/ajr.12878

**Published:** 2022-05-21

**Authors:** Jonathan Souter, James A. Smith, Kootsy Canuto, Himanshu Gupta

**Affiliations:** ^1^ Rural and Remote Health NT, College of Medicine and Public Health Flinders University Darwin, NT Australia; ^2^ Menzies School of Health Research Charles Darwin University Darwin, NT Australia

**Keywords:** Aboriginal and Torres Strait Islander males, health equity, health promotion, men's health, Northern Territory, rural and remote health, strengths‐based approach

## Abstract

**Aims:**

To elucidate key considerations for effective health promotion with Aboriginal and Torres Strait Islander males in remote Northern Territory.

**Context:**

Despite the significant disparities in health outcomes amongst Aboriginal and Torres Strait Islander males, particularly in remote Northern Territory, investment in health promotion policy and practice has been inadequate. Progressing towards self‐determination with Aboriginal and Torres Strait Islander males, and to meet the unique health and well‐being needs of this marginalised demographic, consideration for staff retention and training, strengths‐based approaches, and implications of divergent masculinities must be considered when devising and delivering culturally responsive and appropriate health promotion interventions. Health promotion needs to be conducted in a collaborative manner, and in less conventional settings, to better engage Aboriginal and Torres Strait Islander males.

**Approach:**

This commentary draws on the author's reflections about working in remote Aboriginal and Torres Strait Islander health policy, practice and research contexts in Northern Australia. It brings together diffuse strands of scholarship about Aboriginal and Torres Strait Islander male health; Aboriginal and Torres Strait Islander health promotion; and health promotion in rural and remote contexts. In doing so, we identify and discuss strategies that have potential to strengthen Aboriginal and Torres Strait Islander male health promotion in rural and remote Australia.

**Conclusion:**

Health services and professionals in remote Northern Territory must leverage the inherent strengths of Aboriginal and Torres Strait Islander males to imbue service delivery with both meaning and capacity for self‐determination. In doing so, this might ultimately help to alleviate the marginalisation of this demographic.

## 1 INTRODUCTION

Scholarship teems with publications that highlight the significant health disparities Aboriginal and Torres Strait Islander males experience, and the importance of addressing social and cultural determinants of health.^1^, ^2^, ^3^ Yet, the most promising means for addressing such issues, at a practical level, are limited. Health promotion that privileges the knowledges and strengths of Aboriginal and Torres Strait Islander males is one such means to sustainably improving the health inequities faced by this demographic.

Remoteness is a notable predisposing factor to more unfavourable health outcomes and has a cumulative impact on the negative repercussions of colonisation for Aboriginal and Torres Strait Islander males, and subsequent intergenerational trauma.^3^

This commentary aims to identify ways to improve the health and well‐being of Aboriginal and Torres Strait Islander males in remote Australia, particularly in the Northern Territory. Implications for health organisations and professionals based in isolated Aboriginal and Torres Strait Islander settings are discussed. This strengths‐based health promotion discourse is critical for ameliorating the ongoing marginalisation remote Aboriginal and Torres Strait Islander males face.^4^, ^5^, ^6^, ^7^, ^8^, ^9^

## 2 GENDER‐SPECIFIC HEALTH CARE

It is well documented that males are more willing to engage with male health professionals whom they trust.^4^, ^10^ This observation is considerably more pronounced in the context of Aboriginal and Torres Strait Islander males for both socio‐cultural and gender‐specific reasons.^5^ Trust is generally time‐contingent, though the alarmingly high attrition rate of primary health care staff in remote regions of Australia makes this difficult.^11^ According to Zhao et al.,^12^ only 20% of remote area nurses (RANs) and Aboriginal Health Practitioners (AHPs) remain working at the same clinic 12 months after commencing. This means non‐Indigenous health staff have inadequate time to cultivate even rudimentary levels of cultural competence, and basic local language skills. In this context, the perceived reluctance of Aboriginal and Torres Strait Islander males to engage ‘appropriately’ reflects the health system's inability to cater to their needs in a culturally respectful way.

Indigenous language learning and cultural competency training needs to become mandatory for health professionals working in these settings, and not merely an optional extra‐curricular activity. This is critical for enhancing health communication in both clinical and non‐clinical primary health care settings.^13^, ^14^ While we acknowledge that cultural competency training does not necessarily result in culturally safe practice, it offers one strategy to better engage Aboriginal and Torres Strait Islander males ‘on their terms’. In addition, building the capacity of non‐Indigenous health professionals to adequately acknowledge and include divergent masculinities and viewpoints fostered by Aboriginal and Torres Strait Islander males is also important.^15^ Addressing these concerns will strengthen health promotion efforts with Aboriginal and Torres Strait Islander male health in remote Northern Territory.

While Aboriginal Community Controlled Health Services (ACCHSs) are considered to be paragons of culturally sensitive health care delivery, a lot of Aboriginal and Torres Strait Islander males continue to remain apprehensive about utilising ACCHSs.^4^, ^5^, ^10^ It is pivotal that health organisations working with Aboriginal and Torres Strait Islander males in remote Northern Territory exercise more consideration to utilising male AHP's and Aboriginal Health Workers (AHW's) as the central assets they are.^16^ Indeed, achieving this has potential to bolster the cultural and linguistic training of non‐Indigenous male health professionals; improve the retention rate of both non‐Indigenous male health professionals and AHPs working in this setting; and ultimately increase engagement rates of Aboriginal and Torres Strait Islander males.^17^

## 3 BEYOND DEFICIT DISCOURSES AND PATERNALISTIC MODELS OF PRIMARY HEALTH CARE

Discourses pertaining to deficits—absence, lack or failure—of Aboriginal and Torres Strait Islander males pervade health policy and practice.^6^ Similarly, an inherently paternalistic biomedical model dominates the health care landscape of remote Northern Territory, a model renowned for fostering dependency and disempowerment.^18^, ^19^ Together, deficit discourses and reactive models of health care inhibit scope for delivering effective health promotion to Aboriginal and Torres Strait Islander males in remote Northern Territory. Where positive models have been adopted, they have been inadequately resourced and have seldom been sustainable.

Public health policy and practice in the context of remote Northern Territory fundamentally needs to refocus its efforts towards health promotion that is meaningful to remote Aboriginal and Torres Strait Islander males. This can be achieved by leveraging the many strengths of this demographic, a ‘strengths‐based’ approach that illuminates their exquisite flare in the realms of leadership, sport, music and art.^20^ In delivering health promotion interventions to Aboriginal and Torres Strait Islander males through settings that are inherently meaningful to them—such as through sports clubs, and on country experiences like hunting—the engagement of this underrepresented demographic might be significantly bolstered.^21^ This approach has been used successfully in other global men's health promotion contexts.^22^

## 4 DIVERGENT CONCEPTUALISATIONS OF MASCULINITIES

Health professionals working with Aboriginal and Torres Strait Islander males, and the health professionals with which they interact, must have an awareness of the divergent conceptualisations of masculinities; particularly how hegemonic constructions of masculinity might impede their own sense of identity and, ultimately, their health and well‐being.^9^, ^23^ This is paramount in delivering effective health promotion strategies for Aboriginal and Torres Strait Islander males as it resituates Western hegemonic norms, by seeking to privilege Indigenous masculinities, and associated Indigenous knowledges and practices.^24^ Merlino et al.^21^ outline how hegemonic masculinity, which is typified by a toxic degree of stoicism, is correlated with reduced health literacy. Conversely, enabling an alternative and congruent Indigenous masculinity, or hybrid models of Indigenous and Western masculinities, can potentially improve health literacy outcomes for Aboriginal and Torres Strait Islander males.^15^, ^25^

## 5 CONCLUSION

Future health promotion action involving Aboriginal and Torres Strait Islander males, living in remote Australia, needs to be more culturally responsive. It must shift away from an entrenched deficit‐based model of health care to one that is strengths‐based, which celebrates the cultural heritage and contemporary achievements of this marginalised population. Indeed, health services and professionals in remote Northern Territory must leverage the inherent strengths of Aboriginal and Torres Strait Islander males to imbue service delivery with both meaning and capacity for self‐determination Until then, the magnitude of health inequities will remain proportional to the vast chasms of gender‐specific, age‐appropriate and culturally responsive health promotion planning and delivery.

## AUTHOR CONTRIBUTIONS

JS: conceptualization; investigation; methodology; writing – original draft; writing – review and editing. KC: conceptualization; supervision; writing – review and editing. HG: supervision; writing – original draft; writing – review and editing.

## CONFLICT OF INTEREST

We have no conflicts of interest to declare.
